# Emotional pictures and sounds: a review of multimodal interactions of emotion cues in multiple domains

**DOI:** 10.3389/fpsyg.2014.01351

**Published:** 2014-12-01

**Authors:** Antje B. M. Gerdes, Matthias J. Wieser, Georg W. Alpers

**Affiliations:** ^1^Clinical and Biological Psychology, Department of Psychology, School of Social Sciences, University of MannheimMannheim, Germany; ^2^Department of Psychology, University of WürzburgWürzburg, Germany; ^3^Otto-Selz Institute, University of MannheimMannheim, Germany

**Keywords:** multimodal emotion processing, emotional pictures, emotional sounds, audiovisual interactions, emotional scene stimuli, auditory stimuli

## Abstract

In everyday life, multiple sensory channels jointly trigger emotional experiences and one channel may alter processing in another channel. For example, seeing an emotional facial expression and hearing the voice’s emotional tone will jointly create the emotional experience. This example, where auditory and visual input is related to social communication, has gained considerable attention by researchers. However, interactions of visual and auditory emotional information are not limited to social communication but can extend to much broader contexts including human, animal, and environmental cues. In this article, we review current research on audiovisual emotion processing beyond face-voice stimuli to develop a broader perspective on multimodal interactions in emotion processing. We argue that current concepts of multimodality should be extended in considering an ecologically valid variety of stimuli in audiovisual emotion processing. Therefore, we provide an overview of studies in which emotional sounds and interactions with complex pictures of scenes were investigated. In addition to behavioral studies, we focus on neuroimaging, electro- and peripher-physiological findings. Furthermore, we integrate these findings and identify similarities or differences. We conclude with suggestions for future research.

## INTRODUCTION

In daily life, a wide variety of emotional cues from the environment reaches our senses. Typically, multiple sensory channels, for example vision and audition are integrated to provide a complete assessment of the emotional qualities of a situation or an object. For example, when someone is confronted with a dog, the evaluation of its potential dangerousness or friendliness will be more effective if visual (e.g., big vs. small dog; tail wagging or not) and auditory information (growling vs. friendly barking) can be integrated. While some of the information carried in either one of the channels may be redundant, the channels may also interact; i.e., a fierce bark may boost visual attention to the dog’s bared teeth.

Despite the obvious relevance of multimodal perception in everyday life, emotion research has typically only investigated unimodal cues – with an apparent emphasis on visual stimuli. To cope with (a) limited processing capacities within a sensory modality and (b) the need to detect information which is relevant for survival, emotionally relevant cues have been suggested to modulate attention and selectively enhance perception (Vuilleumier, 2005; Pourtois et al., 2012).

Indeed, for the *visual domain* it has been shown that emotional cues – especially with threatening, but also with appetitive content – are preferentially processed in very early sensory areas (Schupp et al., 2003b; Pourtois et al., 2005; Gerdes et al., 2010). Emotional pictures influence perceptual processing and attract enhanced attention (Öhman and Wiens, 2003; Alpers and Pauli, 2006; Alpers and Gerdes, 2007; Gerdes et al., 2008, 2009; Stienen et al., 2011; Pourtois et al., 2012; Gerdes and Alpers, 2014). Furthermore, distinct and intensive behavioral responses, physiological reactions, and brain activations are robustly evoked by emotional pictures (e.g., Lang et al., 1998; Neumann et al., 2005; Alpers et al., 2011; Eisenbarth et al., 2011; Plichta et al., 2012). According to Lang (1995) and Lang et al. (1998), the emotional response system is founded on an appetitive and defensive motivational system. Emotional states reflect these basic motivational systems and can be described in terms of affective valence and arousal. For example, a number of physiological measures are shown to covary with the valence or arousal of emotional cues: electromyographic (EMG) activity, heart rate responses and the startle reflex were shown to be sensitive to valence, whereas skin conductance and slow cortical responses are more sensitive to arousal (for more elaborative reviews on the processing of emotional pictures see, e.g., Bradley and Lang, 2000b; Brosch et al., 2010; Sabatinelli et al., 2011). Generally, enhanced processing gains of emotional cues may help individuals to quickly initiate adequate approach or avoidance behavior and therefore increases the chance of survival or well-being (Lang et al., 1997).

On the neural level the amygdala has long been identified as a key structure of emotional detection both in humans and animals (for reviews and meta-analysis see LeDoux, 2000; Phan et al., 2002; Costafreda et al., 2008; Armony, 2013). Relevant for the present context, via the thalamus the amygdala receives input not only from the visual modality but from all senses (Nishijo et al., 1988; Amaral et al., 1992; Amaral, 2003). The amygdala is instrumental in the relevance detection for biologically relevant cues and has been documented to operate independently from the sensory modality which conveyed the information (Armony and LeDoux, 2000; Sander et al., 2003; Zald, 2003; Öhman, 2005; Stekelenburg and Vroomen, 2007; Scharpf et al., 2010; Armony, 2013). There is empirical evidence that the amygdala processes, e.g., emotional visual (Royet et al., 2000; Phan et al., 2002) and auditory cues (Fecteau et al., 2007; Klinge et al., 2010), as well as olfactory (Gottfried et al., 2002), and gustatory cues (O’Doherty et al., 2001).

Despite the evidence that visual and auditory emotion processing recruits similar brain structures, research on emotional auditory information and on multimodal cues is relatively scant. Until recently, this research field has mainly examined multimodal integration in social communication, i.e., face–voice stimuli (for a recent review see Klasen et al., 2012).

## FACE–VOICE INTERACTION

From studies using combined face-voice stimuli, we know that audiovisual integration can facilitate and improve perception, even beyond the emotion effects within each separate channel. Emotion recognition is improved in response to multimodal compared to unimodal face-voice stimuli (Vroomen et al., 2001; Kreifelts et al., 2007; Paulmann and Pell, 2011). Furthermore, the identification of an emotional facial expression is facilitated when the face is accompanied by an emotional congruent voice and the evaluation of emotional faces is biased toward the valence of a simultaneously presented voice (de Gelder and Vroomen, 2000; de Gelder and Bertelson, 2003; Focker et al., 2011; Rigoulot and Pell, 2012). Such interactions appear to be independent of attentional allocation, i.e., even when participants are instructed to pay attention to only one sensory modality, emotional information of a concurrent but non-attended sensory channel influences the processing of the attended modality (Collignon et al., 2008). Likewise, if emotional faces and voices tap the same emotional valence (emotional congruency), they were processed faster than emotionally incongruent stimulus pairs or unimodal stimuli even when the attentional focus was explicitly directed to the faces or to the voices (Focker et al., 2011). Furthermore, this cross-modal influence was independent of a demanding additional task which had to be performed in parallel (Vroomen et al., 2001).

On the neuronal level, face–voice integration can occur at early perceptual stages of stimulus processing (for more specific information see the review of Klasen et al., 2012). Furthermore, specific brain areas such as superior and middle temporal structures and the fusiform gyrus, as well as parts of the emotion processing network including the thalamus, amygdala, and insula are consistently involved in emotional face–voice integration (see also Klasen et al., 2012). Taken together, the integration of emotional faces and voices is an important part of social interaction and the prioritized processing and early integration of emotional face–voice pairs is an essential feature of social cognition (de Gelder and Vroomen, 2000).

## BEYOND EMOTIONAL “FACE–VOICE” PROCESSING

In this review, we argue that a broader variety of stimuli should be considered in audiovisual emotion processing. While stimuli which are directly linked to human communication may represent an important subset of cues, a meaningful extension to existing concepts of multimodality should be carried forward by considering other domains as well. In this review we focus on visual and auditory cues across a wide range of semantic categories.

We start out with a short overview of studies mainly from the visual domain which focus on differences between emotional human communication and scene processing. This section demonstrates that the processing of communication vs. scene stimuli recruits different brain structures and elicit distinct electro- and peripher-physiological responses. Thus, we argue that a distinction between those kinds of stimuli in multimodal emotion research is important and useful.

Because research on emotional sounds is much less frequent than research on emotional pictures we give a short overview of how emotional complex sounds can affect self-report ratings, physiological responses, and brain processes, and then summarize similarities and differences between emotional sound and picture processing.

Furthermore, we review studies which investigate how emotional information in one sensory modality can influence information processing of neutral cues in another sensory modality. Finally, we will summarize the existing studies that focus on interactions of the concurrent processing emotional visual and emotional auditory cues beyond faces and voices. We will conclude with a short summary and an outlook on research questions where the application of multimodal stimuli is particular interesting.

## HUMAN COMMUNICATION VS. EMOTIONAL SCENE STIMULI

Beyond face–voice integration, there are only few studies which focus on audiovisual interactions in emotion research. On the one hand, quite similar to face–voice interactions, some studies investigated multimodal integration in human communication with regard to bodily gestures and vocal expressions (Stekelenburg and Vroomen, 2007; Jessen and Kotz, 2011; Stienen et al., 2011; Jessen et al., 2012). On the other hand, there are several studies which examine influences of music on the processing of visual stimuli (e.g., Baumgartner et al., 2006; Logeswaran and Bhattacharya, 2009; Marin et al., 2012; Hanser and Mark, 2013; Arriaga et al., 2014). Because music is man-made, many theorists claim, that it is in essence another form of human communication. Therefore, music may be more similar to the communicative channels described above than to other naturally occurring sounds. In a similar vein, it has been argued that music has no obvious survival value (Juslin and Laukka, 2003).

Generally, it has been demonstrated that a fast and effective integration of stimuli across different modalities is necessary in several (survival-relevant) contexts. These contexts are certainly not limited to social situations. Examples for non-social situations of high biological significance may be a growling bear, swirling wasps, or an approaching thunderstorm. In these examples congruent visual and auditory information is transmitted (to the individual), whose prioritized processing may help the organism to survive. The first obvious advantage of multimodal information is that uncertainties in one sensory channel can be easily compensated and complemented by the other channel. In addition, even if the (emotional) information conveyed by ear and eye is obviously redundant, multisensory integration effects can be clearly distinguished from redundancy effects within one modality. Again, the only empirical evidence supporting this claim stems from research on face–voice pairings. Redundant emotional information within the same modality leads to (post-) perceptual interferences shown by a lesser accuracy and longer response latencies within an emotional expression discrimination task. In contrast, congruent information of faces and voices was integrated early and pre-attentive with a clear perceptual benefit (Pourtois and Dhar, 2013). Thus, both senses supplement each other to create a distinct multisensory emotional percept (for a detailed discussion on stimulus redundancy; Pourtois and Dhar, 2013). Compared to information from face and voice, in many natural situations, not only concordant but also unrelated information during the same event can be conveyed by the different sensory channels (e.g., the sound of an emergency siren while watching children playing on a playground).

Empirical evidence for multifaceted differences between human communication and scene stimuli comes from the visual domain. On the peripher-physiological level, Alpers et al. (2011) showed that emotional scenes and faces were rated similarly, but the pattern of physiological responses measured by startle reflex, heart rate, and skin conductance was different. Startle responses to emotional scenes were modulated by valence with lowest amplitudes for positive, intermediate for neutral, and highest amplitudes for negative scenes, whereas the startle response was similarly enhanced in response to negative and positive faces. Furthermore, negative scene picture show a greater heart rate deceleration compared to neutral and positive scene; whereas negative and positive faces were followed by heart rate deceleration. These results indicate that scenes result in a valence based modulation, faces an arousal based modulation. In contrast, the skin conductance was arousal-modulated for the scene pictures with higher responses to the negative pictures and a valence-specific modulation for the faces with highest responses to positive faces. The facial EMG showed similar responses to both contents, but responses were slightly greater in response to scenes. Likewise comparing emotional faces and scenes, a recent study (Wangelin et al., 2012) showed that emotional scenes evoked stronger reactions in autonomic, central, and reflex measures in comparison to faces. In a meta-analysis (Sabatinelli et al., 2011), it was shown that emotional scenes elicited activation in occipital regions, the pulvinar, and the medial dorsal nucleus of the thalamus whereas the fusiform gyrus and the temporal gyrus were specifically activated in response to faces. Thus, measured emotion effects can strongly depend on the presented class of stimuli. Particularly, in emotion research one may argue that while face stimuli certainly convey emotional information, they do not necessarily elicit emotions in the observer (Ruys and Stape, 2008). Taken together, evidence from the visual domain clearly highlights the importance of a separate consideration of face and scene stimuli in emotion research. Analogous to that, for the auditory domain there is empirical evidence that human vocal and non-vocal sounds generally have different electrophysiological correlates and can elicit distinct responses in auditory regions (Meyer et al., 2005; Bruneau et al., 2013).

Thus, investigations with affective scene cues which have been consistently demonstrated to elicit emotional responses on behavioral and physiological levels (for emotional sounds see below) are needed to answer research questions about multi-modal emotion processing beyond face-voice stimuli.

## EMOTIONAL SOUND PROCESSING

Compared to visual cues, sounds are still investigated only rarely. It is likely that this is due to the development of research traditions according to practical considerations rather than a reflection of the relative importance of auditory cues. Compared to pictures, sounds may be somewhat less amenable to experimental designs in the laboratory. However, sounds can clearly prompt strong emotional responses as has been shown in a large internet-based survey (Cox, 2008b). The development of the International Affective Picture System (IAPS; Lang et al., 2008) has been followed by a similar collection of sounds, the International Affective Digitized Sounds (IADS; Bradley and Lang, 2007) – a series of naturally occurring human, non-human, animal, and environmental sounds (e.g., bees buzzing; applause, explosions). Existing research on emotional sound processing (beyond voices) has been almost exclusively used this series as stimulus material (see below). In two experiments by Bradley and Lang (2000a), it was shown that valence and arousal ratings of these sounds were comparable to affective pictures from the IAPS. Furthermore, emotionally arousing sounds were also remembered better than neutral sounds in a free recall task. On a physiological level, emotionally arousing sounds elicit larger electrodermal activity which is generally known to be sensitive to the arousal of emotional stimuli (Bradley and Lang, 2000a). In comparison to pleasant sounds, the startle response to unpleasant sounds is enhanced and unpleasant sounds were accompanied by stronger corrugator activity and larger heart rate deceleration. This suggests that unpleasant sounds reliably activate the defensive motivational system (Bradley and Lang, 2000a). Another study showed that emotional sounds were accompanied by larger pupil dilatation which is an index of higher autonomic activity elicited by emotion (Partalaa and Surakka, 2003). Electrophysiological results suggest that aversive auditory cues (as, e.g., squeaking polystyrene) compared to neutral sounds were accompanied by a more pronounced early negativity and later positivity of event-related brain potentials as a measure of enhanced allocation of attention (Czigler et al., 2007) similar to what has been observed in emotional pictures (Schupp et al., 2003a). In contrast, unpleasant environmental sounds capture enhanced attention (shown by increased P3a amplitudes) but do not influence earlier components of perceptual processing (Thierry and Roberts, 2007). Similarly, two fMRI studies (Scharpf et al., 2010; Viinikainen et al., 2012) measured brain activation in response to emotional sounds from the IADS. Both studies showed that emotional sounds elicited strong activation in the amygdala compared to neutral sounds. Specifically, Viinikainen et al. (2012) showed that there was a quadratic U-shaped relationship between the sound valence and brain activation in the medial prefrontal cortex, auditory cortex, and amygdala with the weakest activation for neutral and increased activation for unpleasant and pleasant sounds.

Importantly, in an fMRI-study (Kumar et al., 2012) there was evidence that the amygdala encodes both the acoustic features of an auditory stimulus and the perceived unpleasantness. Specifically, acoustic features modulate effective connectivity from auditory cortex to the amygdala whereas valence modulates the effective connectivity from amygdala to the auditory cortex. Thus, control of acoustic features is of specific importance in research on emotional sounds.

A recent study from our research group investigated the processing of emotional sounds from the IADS within the auditory cortex (Plichta et al., 2011). Because fMRI scanner noise can interfere with auditory processing we used near-infrared spectroscopy (NIRS) which is a silent imaging method. In addition, the sound material was carefully controlled for several physical parameters such as loudness and spectral frequency. Unpleasant and pleasant sounds enhanced auditory cortex activation as compared to neutral sounds suggesting that the enhanced activation of sensory areas in response to complex emotional stimuli is apparently not restricted to the visual domain.

Further support for this observation comes from an MEG-Study investigating the influence of emotional content of complex sounds on auditory-cortex activity, both during anticipation and hearing of emotional and neutral sounds (Yokosawa et al., 2013). Indeed, during the hearing as well as during the anticipation period, unpleasant and pleasant sounds evoked stronger responses within the auditory cortex than neutral sounds.

In sum, there is now considerable evidence that complex highly arousing pleasant and unpleasant sounds are processed more intensively on a peripheral as well as on early sensory processing levels. Thus, using standardized emotional sounds (e.g., the IADS) can serve as a useful research tool to elicit emotions and investigate emotion processing.

## EMOTIONAL SOUNDS AND PICTURE PROCESSING: SIMILARITIES AND DIFFERENCES

Generally, emotional sound and picture processing is very comparable. The pattern of behavioral and physiological and electrophysiological reactions elicited by emotional sounds is comparable to emotional pictures (Bradley and Lang, 2000a; Schupp et al., 2003a; Czigler et al., 2007). However, there is some evidence that reactions to emotional sounds are weaker (Bradley and Lang, 2000a) and occur later (Thierry and Roberts, 2007). On the neuronal level, both emotional sounds and pictures gain privileged access to processing resources in the brain. Brain responses to visual, auditory, and olfactory stimuli were measured with PET showing for all three modalities, that all emotional stimuli activated the orbitofrontal cortex, the temporal pole, and the superior frontal gyrus (Royet et al., 2000). In addition, Scharpf et al. (2010) compared brain activation to sounds with responses to IAPS pictures. Independent of the sensory modality, the amygdala, the anterior insula, the STS, and the OFC showed increased activation during the processing of emotional as well as social stimuli. Also comparing brain activation to emotional pictures from the IAPS and to emotional sounds from the IADS, increased amygdala activity in response to both, emotional pictures and sounds were reported (Anders et al., 2008). Differentially, the left amygdala was sensitive to the valence of pictures and negative sounds whereas the right amygdala responded to the valence of positive pictures. A recent study directly aimed at investigating whether affective representations differ with sensory modality (Shinkareva et al., 2014). Therefore, emotional picture and sound stimuli were presented in an event-related fMRI experiment. The results mainly provide evidence for a modality specific instead of a modality-general valence processing effect. Specifically, voxels were identified that were sensitive to the valence of pictures within the visual modality, as well as voxels that were sensitive to the valence of sounds within the auditory modality, but no voxels that were sensitive to valence across the two modalities.

To sum up, emotional pictures and sounds mainly elicit similar reactions on the level of self-report, behavioral, physiological, and neuronal – both types of stimuli strongly activate appetitive and defensive motivational circuits (Bradley and Lang, 2000a; Lang and Bradley, 2010). The reported processing differences (e.g., intensity of reaction, laterality effects, and timing) might be – at least partly – the result of methodological differences and different stimulus characteristics which are obvious between sounds and pictures (e.g., the dynamic nature of sounds). Thus, to account for such differences and to interpret potential processing differences, systematic and direct comparisons between emotional picture and sound processing with well controlled and (physically) comparable stimuli (e.g., conditioned stimuli) are urgently needed.

## AUDIO–VISUAL INTERACTIONS

### INTERACTION OF VISUAL AND AUDITORY PROCESSING

Generally and beyond the emotional domain, it is well established that visual information can foster early stages of auditory processing and vice versa. For example, auditory speech perception can be strongly influenced by the viewing of visual speech stimuli on the perceptual (McGurk and MacDonald, 1976) as well as on the neuronal level (see, e.g., Kislyuk et al., 2008). Likewise, visual processing can be strongly altered by concurrent sounds even at the earliest stage of cortical processing (Bulkin and Groh, 2006; Shams and Kim, 2010). Based on these findings it seems plausible that such interaction may also occur when emotional information is conveyed by (at least) one of the sensory channels. Indeed, a small but growing number of studies suggest that (emotion) processing in the auditory system can be influenced by (non-related) emotional information coming from the visual modality and vice versa (see below).

### INTERACTION OF EMOTIONAL VISUAL AND NON-EMOTIONAL AUDITORY PROCESSING

On a behavioral level, a recent study investigated the influence of emotional IAPS pictures on the classification of high and low pitch tones but did not find an effect of picture valence on the auditory classification (Ferrari et al., 2013).

On the physiological level, it is well-known that emotional visual stimuli can modulate the acoustic startle reflex elicited by loud, abrupt, and unexpected sounds: negative pictures enhance, positive pictures dampen the blink magnitude in response to the unexpected sound (e.g., Lang et al., 1990). Moreover, the electrocortical response to the acoustic startle probe (P3 component) was also found to be modulated by the arousal of the emotional pictures in the foreground with smaller amplitudes for high arousing pictures (Keil et al., 2007).

Regarding electrophysiological responses, the presentation of unpleasant pictures has a significant impact on event-related potentials of the EEG to strongly deviant tones. During the presentation of unpleasant pictures, high deviant tones elicited larger N1 and P2 responses than during the presentation of pleasant pictures which was interpreted as a sensitization to potentially significant deviant events (N1) and enhanced attention (P2) to regular external events (Sugimoto et al., 2007). Similarly, auditory novelty processing was enhanced by negative IAPS pictures. Participants had to judge (emotional) picture pairs as equal or different while ignoring task irrelevant sounds. During the presentation of negative IAPS-pictures, novel sounds compared to the standard tone provoked enhanced distraction effects shown on the behavioral level as well by the modulation of event-related potentials (enhanced early and late novelty P3; Dominguez-Borras et al., 2008a,b). In a similar vein, pleasant pictures were shown to modulate auditory information processing such that they significantly attenuated the Mis-Match-Negativity (MMN) in response to a change within an auditory stimulus stream. Thus, pleasant pictures can be seen as a kind of safety signals and probably reduce the need for auditory change detection (Surakka et al., 1998).

Further support for crossmodal influences of emotion is provided by a MEG study showing that unpleasant pictures diminish auditory sensory gating in response to repeated neutral tones as an index of neuronal habituation (Yamashita et al., 2005). Presenting neutral tones subsequent to emotional pictures, another study showed that neutral tones prompted larger ERP amplitudes (N1 and N2) when emotional relative to neutral pictures were presented before, indicating enhanced enhance attention and orienting toward neutral tones encoded in the context of emotional scenes (Tartar et al., 2012). All studies reported above indicate that emotional visual information can enhance auditory processing. However, the question arises whether increasing demands of an auditory task might interfere with the processing of emotion in the visual domain. This may be due to competition for limited processing resources, a process which has been documented for competing emotional pictures within the same modality (Schupp et al., 2007). However, the processing of emotional IAPS pictures was not modulated by an additional auditory detecting task with increasing complexity (Schupp et al., 2008). Thus, emotion processing in the visual domain was not affected by task demands in the auditory modality. This finding is in line with the multiple resource theory which assumes that each sensory modality has separate pools of (attentional) resources (Wickens, 2002).

Taken together, emotional cues in the visual domain are able to enhance concurrent as well as subsequent auditory processing even at very early processing stages with no or low costs for emotion processing.

### INTERACTION OF EMOTIONAL AUDITORY AND NON-EMOTIONAL VISUAL PROCESSING

To the best of our knowledge, evidence that emotion cues from the auditory modality can also influence non-emotional (basic) visual information processing is nearly missing. One study investigated the influence of emotional sounds on visual attention in a spatial cueing paradigm (Harrison and Davies, 2013). Here, non-speech environmental sounds from the IADS were presented spatially matched to the locations of subsequent visual targets. Indeed, results show for right-sided targets that neutral and positive sounds elicited faster responses to valid trials (where the sound and the visual target were presented on the same side) compared to invalid trials. In contrast, after negative sounds, the reaction time to valid trials was slower suggesting faster attentional disengagement from negative sounds.

Another study used spoken emotional and neutral words that were followed by a visually presented neutral target word (Zeelenberg and Bocanegra, 2010). It was found that identification of a masked visual word was improved by preceding spoken emotional words as compared to neutral ones. These findings can be interpreted as first evidence that affective sounds may influence at least subsequent visual (word) processing. However, much more studies are needed in which the effects of emotional sounds on concurrent and subsequent visual processing are investigated.

### INTERACTION OF EMOTIONAL AUDITORY AND EMOTIONAL VISUAL PROCESSING

We assume that audio–visual interactions are possible in both directions. As reviewed above, emotional visual as well as auditory information are preferentially and intensively processed. Thus, one can expect that audio–visual interactions of emotional stimuli occur even stronger if emotional information is conveyed by *both* modalities. Regarding that question, self-report data of an internet-based survey suggest that sound stimuli were significantly perceived as more horrible when they were accompanied by pictures that show associated information (e.g., the sound of a crying baby combined with a picture of a crying baby) compared to pictures with unassociated pictures (Cox, 2008a). Using affective IADS sounds; Scherer and Larsen (2011) found significant cross-modal priming effects for negative sound primes on emotional visual word targets. Experimentally, self-report (valence and arousal) and physiological variables were measured in response to unimodal and bimodal presented emotional sounds and pictures in a within subjects design. Unpleasant and pleasant stimuli had similar effects on self-report, heart rate, heart rate variability, and skin conductance with no effect of stimulus modality. Contrary to expectations, bimodal presentation with congruent visual and auditory stimuli did not enhance the effects (Brouwer et al., 2013).

In a similar vein, we recently conducted an EEG-study in which unpleasant, pleasant, and neutral IAPS pictures were preceded by unpleasant, pleasant, and neutral IADS sounds. Ratings and electrophysiological data suggest that (emotional) sounds clearly influence emotional picture processing (Gerdes et al., 2013). We could demonstrate that audiovisual pairs with pleasant sounds and pictures were rated as more pleasant than pleasant pictures only. In addition, valence congruent audiovisual combinations were rated as more emotionally as other incongruent combinations. Electrophysiological measures showed that ERP amplitudes (P and P2) were enhanced in response to all pictures which were accompanied by emotional sounds compared to pictures with neutral sounds. These findings can be interpreted as evidence that emotional sounds may unspecifically increase sensory sensitivity or selective attention (P1, P2) to all incoming visual stimuli. Most importantly, unpleasant pictures with pleasant sounds prompted larger ERP amplitudes (P1 and P2) compared to unpleasant pictures with unpleasant sounds. The reduced amplitudes in response to congruent sound-picture pairs suggest that the processing of unpleasant pictures is facilitated (i.e., less processing resources are needed) when they were preceded by congruent unpleasant sounds.

Taken together, the above mentioned studies strongly suggest that emotion processing in one sensory modality can strongly affect emotion processing of another modality during very early stages of neuronal processing, as well as on the self-report level.

For a short overview of the here reviewed studies see **Table 1**.

**Table 1:** Overview of the reviewed studies (in alphabetical order) mainly investigating (1) emotional sound processing, interaction of (2) emotional visual and non-emotional auditory processing, (3) emotional auditory and non-emotional visual processing, (4) emotional auditory and emotional visual processing with information about the used stimuli, the dependent variables and a short summary of the main result.

Study	Auditory stimuli (Valences)	Visual stimuli (Valences)	Dependent variables	Main result
**1) Emotional sound processing**				
Anders et al. (2008)	IADS (NEG/NEU/POS)	IAPS (NEG/NEU/POS)	Brain activation (fMRI); startle response (EMG), SCR; rating	Amygdala activation: left amygdala is sensitive to valence of pictures and negative sounds; right amygdala is sensitive to valence of positive pictures.
Bradley and Lang (2000a)	IADS (NEG/NEU/POS)		Startle response (EMG), facial EMG; HR deceleration, SCR, free recall, Rating	Startle response/Corrugator EMG/HR deceleration: NEG > NEU SCR/Free recall: NEG/POS > NEU Rating: Valence: POS > NEU > NEG Arousal: NEG > = POS > NEU
Cox (2008b)	Human and environmental sounds^a^ (NEG)		Rating (internet survey)	Most aversive sound: sound of someone vomiting
Czigler et al. (2007)	scraping sounds, environmental sounds^c^ (NEG/NEU)		Oddball ERPs (EEG), RT, Hit Rate	ERPs: time range 154–250 ms: NEG > NEU Time range 372–456 ms: NEG > NEU
Kumar et al. (2012)	Scraping sounds, animal cries, environmental sounds^c^ (NEG/NEU)		Brain activation (fMRI)	Amygdala encodes both the acoustic features of a stimulus and its valence.
Partalaa and Surakka (2003)	IADS (NEG/NEU/POS)		Pupil size, rating	Pupil size: NEG/POS > NEU Rating: valence: POS > NEU > NEG Arousal: NEG > = POS > NEU
Plichta et al. (2011)	IADS (NEG/NEU/POS)		Brain activation (fNIRS); rating	Auditory cortex activation: NEG/POS > NEU
Royet et al. (2000)	Environmental sounds and vocalizations^c^ (NEG/NEU/POS)	complex scenes^c,1^ (NEG/NEU/POS)	Brain activation (PET)	*Pictures and sounds:* activation of orbitofrontal cortex, the temporal pole, and the superior frontal gyrus, in the left hemisphere: NEG/POS > NEU *Pictures*: activation of hypothalamus and the subcallosal gyrus: NEG/POS > NEU
Scharpf et al. (2010)	IADS (NEG/NEU/POS)	IAPS (NEG/NEU/POS)	Brain activation (fMRI)	*Pictures and sounds*: activation of amygdala, the anterior insula, the STS, and the OFC: NEG/POS > NEU
Shinkareva et al. (2014)	IADS (NEG/POS)	IAPS (NEG/POS)	Brain activation (fMRI), Rating	Significant sensitivity of voxels within sensory cortex regions to valence within modality, but not across modalities.
Thierry and Roberts (2007)	Environmental sounds^b^ (NEG/NEU)		ERPs (EEG), detection rate	ERPs (P3a): NEG > NEU
Viinikainen et al. (2012)	IADS (NEG/NEU/POS)		Brain activation (fMRI)	Medial prefrontal cortex, auditory cortex, and amygdala activation: NEU < POS/NEG
Yokosawa et al. (2013)	IADS (NEG/NEU/POS)		Brain activation (MEG)	Anticipation of emotional sounds evoked typical MEG (N100 m) responses in the auditory cortex.
**2) Interaction of emotional visual and non-emotional auditory processing**				
Bradley et al. (1990)	White noise (NEU)	IAPS (NEG/NEU/POS)	Startle response (EMG), Rating	Startle response: NEG > NEU > POS
Dominguez-Borras et al. (2008a) and Dominguez-Borras et al. (2008b)	Tones (NEU)	IAPS (NEG/NEU/POS)	ERPs (EEG); RT	ERPs amplitudes (late novelty P3) to novel sounds were enhanced during the presentation of negative pictures.
Keil et al. (2007)	White noise (NEU)	IAPS (NEG/NEU/POS)	ERPs (EEG) Startle response (EMG),	ERPs (P3) to startle probes: NEU > POS/NEG for sound and picture foregrounds.
Schupp et al. (2008)	Tones (NEU)	IAPS (NEG/NEU/POS)	ERPs (EEG)	No influence of auditory task on (emotional) picture processing.
Sugimoto et al. (2007)	Tones (NEU)	IAPS (NEG/NEU/POS)	ERPs (EEG)	High deviant tones elicited larger ERP amplitudes (N1) while viewing negative compared to positive pictures. ERP amplitudes (P2) to standard tones were smaller while viewing positive compared to negative and neutral pictures.
Surakka et al. (1998)	Tones (NEU)	IAPS (NEG/NEU)	ERPs (EEG); Reaction times	Attenuated Mismatch Negativity to tones (MMN) by positive, low arousing pictures.
Tartar et al. (2012)	Tones (NEU)	IAPS (NEG/NEU)	ERPs (EEG)	Early attention and orienting effects to subsequent tones (enhanced N1 and N2) during negative pictures compared to neutral.
Yamashita et al. (2005)	Click tones (NEU)	IAPS (NEG/NEU/POS)	Brain activation (MEG)	P50 m suppression (sensory gating): NEG < NEU
**3) Interaction of emotional auditory and non-emotional visual processing**				
Ferrari et al. (2013)	Tones (NEU)	IAPS and public domain pictures^c^ (NEG/NEU/POS)	RT in a auditory classification task	No influence of emotional pictures on audiovisual interaction. Emotional pictures (POS/NEG) reduce RT in auditory classification task.
Harrison and Davies (2013)	IADS (NEG/NEU/POS)	Target dots (NEU)	RT to the visual target; Rating	No influence of emotional sounds on visual task.
Zeelenberg and Bocanegra (2010)	Spoken words (NEG/NEU)	Target words (NEU)	Word identification	Auditory emotional compared to neutral cues improve visual word identification.
**4) Interaction of emotional auditory and emotional visual processing**				
Brouwer et al. (2013)	IAPS (NEG/POS)	IADS (NEG/POS)	HR, HR Variability, SCR, Rating	*Pictures and Sounds*: HR: POS > NEG HR variability; SCR POS/NEG > NEU No differences between pictures and sounds. No differences between uni- and bimodal stimuli.
Cox (2008a)	Human & environmental sounds^a^ (NEG/NEU)	Pictures^c^ (NEG/NEU)	Rating (internet survey)	Pictures affected sound rating.
Gerdes et al. (2013)	IADS (NEG/NEU/POS)	IAPS (NEG/NEU/POS)	ERPs (EEG), Rating	Significant influences of emotional sounds on visual (emotion) processing.
Scherer and Larsen (2011)	IADS (NEG/POS)	Words (NEG/POS)	Rating; Word evaluation	Significant cross-modal priming effects only for negative primes.


*^a^Sounds selection based on a voting database; ^b^self-recorded sounds; ^c^self-collected stimuli.^1^Additional stimuli: odorants.*

*EEG = electroencephalography; EMG = electromyography; ERPs = event-related potentials; fMRI = functional magnetic resonance imaging; fNIRS = functional near-infrared spectroscopy; HR = heart rate; IADS = International Affective Digitized Sounds (Bradley and Lang, 2007); IAPS = International Affective Picture System (Lang et al., 2008); MEG = magnetoencephalography; NEG = negative stimuli; NEU = neutral stimuli; OFC = orbitofrontal cortex; POS = positive stimuli; RT = (manual) reaction time; SCR = skin conductance response; STS = sulcus temporalis superior.*

## CONCLUSION AND FUTURE DIRECTIONS

As the review summarized at the beginning, the majority of research on unimodal auditory emotion processing provides clear evidence that complex emotional auditory stimuli (mainly investigated with IADS) elicit similarly intensive emotional reactions on behavioral, physiological, and on neuronal levels as traditionally used complex visual emotional scenes. Furthermore, emotional cues from both modalities guide selective attention and receive enhanced processing. This preferential processing can even alter (emotional) information processing in other sensory channels. Specifically, there is evidence that the processing of complex emotional information in one sensory modality can strongly affect (emotion) processing of another modality during very early stages of neural processing, as well as self-reported emotions, and that these effects are bidirectional. As hitherto existing research mainly focused on stimuli of human communication such as faces and voices, the here reviewed work expands the concept of multimodality to a broader variety of human, animal, and environmental cues. However, as this review also indicates research in this field is in its infancy. Consequently, to carve out similarities and differences between the different classes of stimuli, disentangling emotional from social relevance (see, e.g., Bublatzky et al., 2014b), and the impact on audiovisual combinations are promising areas of future research.

From a methodological viewpoint, complex scene stimuli additionally offer the opportunity to separate effects of semantic (or contextual) and emotional (in)congruence in multimodality which is usually confounded in face–voice pairings. Similarly important is the dissociation of (task) difficulty from incongruence in multimodal emotion integration (Watson et al., 2013). Furthermore, effects of presentation order and timing of the stimuli should be investigated systematically, because such methodological differences seem partly responsible for inconsistent findings (see Jessen and Kotz, 2013). Also important, research on multimodality should strongly reveal effects simply caused by stimulus redundancy or by intensity amplification in contrast to inherent multimodality (Pourtois and Dhar, 2013). In order to investigate the (interactive) effects of sounds, more diverse stimulus sets would be highly welcome. Specifically, research on influences of emotional sounds on (subsequent) visual processing is pending. To account for physical differences of emotional picture and sounds, investigations with well controlled and (physically) comparable stimuli (e.g., with instructed fear or conditioning procedures) are urgently needed (see, e.g., Bröckelmann et al., 2011; Bublatzky et al., 2014a).

Regarding the impact of attention and automaticity, there has been a controversy for unimodal emotional cues whether they are processed outside of explicit attention or whether attentional disengagement can reduce neural responses and behavioral output (Pessoa, 2005). For example some studies claimed that visual threat cues activate the amygdala independently from attentional allocation (Straube et al., 2006) or that attentional distraction actually resulted in reduced activation (Pessoa et al., 2002; Alpers et al., 2009). We are not aware of systematic investigations of multimodal emotional stimulation and variations in attention to one or multiple channels. Interestingly, multimodal presentations may provide a particularly fruitful avenue for this debate because it is possible to attend to one channel and ignore the other.

With regards to other electrophysiological indices of preferential processing and attention, the N2Pc or steady-state visual evoked potentials (ssVEPS) which are established in the visual domain (Wieser et al., 2012b; Weymar et al., 2013), would lend themselves for the examination of sound as well as for audio–visual cues and interactions. For example, it would be interesting to see how the N2pc as an index of visual–spatial attention to a salient stimulus in visual search paradigms is modulated by concurrent (emotional) sounds. Also, the influence of emotional sounds on sustained attentional processes (as measured by ssVEPS) would be an interesting research question. The use of different paradigms would help inform us about the different stages at which presumably emotional cues from different modalities interact. Recently, novel paradigms have been introduced to examine the behavioral output of preferentially processed emotional cues (see, e.g., Pittig et al., 2014). If integrated multimodal cues result in a more intensive emotional experience (and neural processing), this may also result in more pronounced behavioral consequences.

To make research more ecological valid and to evolve a broader and more complete concept of emotional multimodality, future research should not only concentrate on audiovisual emotion processing but should also incorporate cues from other sensory channels as, e.g., olfactory (Pause, 2012; Adolph et al., 2013), somatosensory (Francis et al., 1999; Gerdes et al., 2012; Wieser et al., 2012a, 2014), or gustatory signals (O’Doherty et al., 2001; Tonoike et al., 2013) which are also known to elicit emotional reactions and may interact with information processes of other modalities.

Another issue that is of great theoretical and practical importance is the consideration of different populations in the context of multimodal emotion processing. From a clinical perspective, the consideration of multimodal emotional processing is promising for the understanding of several mental disorders (e.g., anxiety disorders). Here, contextual (multimodal) information contributes to the acquisition and maintenance of the disorder (Craske et al., 2006). Accordingly, it has been argued elsewhere that for example in research on social anxiety disorder a crossmodal perspective may help to gain a more complete and ecological picture of cognitive biases and understand fundamental processes underlying biases in social anxiety (Peschard et al., 2014). Altogether, explicit knowledge on multimodal integration and interaction processes can improve the understanding of emotion processing (deficits) and consequently may help to optimize therapeutic approaches (see Taffou et al., 2012; Maurage and Campanella, 2013).

For the most part, the here reviewed interactions between emotional stimuli in the two senses can be explained on the background of the motivational priming theory (Lang, 1995). According to that theory, emotion is considered to be organized around two motivational systems, one appetitive, and one defensive. These systems have evolved to mediate behavior that either promote or threaten physical survival (Lang et al., 1997).

Independent of the sensory modality, emotional information is thought to activate the appetitive or defensive motivational system. Consequently, the engaged motivational system modulates other (brain) processing operations which means that (perceptual) processing of other emotional information can be facilitated or inhibited. These modulatory effects are shown crossmodally, thus, there also seem to be independent of the stimulus modality (see, e.g., Bradley et al., 1990; Lang et al., 1998).

Taken together, the motivational priming theory is able to explain audiovisual *interactions* of emotional information. However, the motivational priming theory does not make any assumptions of how multisensory emotional inputs are combined and integrated. Actually, no specific model exists which accounts for the integration of multisensory emotional information. Generally, one can assume that multisensory integration of emotional information follows similar principles as multisensory integration of other types of complex information (see, e.g., Stein and Meredith, 1993; de Gelder and Bertelson, 2003; Ernst and Bülthoff, 2004; Spence, 2007). Within the scope of the motivational priming theory, motivated attention might influence the efficiency of this integration processes. However, the development and the systematic testing of a specific theoretical framework for multimodal emotion processing is definitely one of the next important future challenges.

## Conflict of Interest Statement

The authors declare that the research was conducted in the absence of any commercial or financial relationships that could be construed as a potential conflict of interest.
